# Psychiatrists’ experiences with the implementation of safewards and other quality improvement work: an explorative, qualitative interview study

**DOI:** 10.1186/s12888-025-07058-x

**Published:** 2025-06-11

**Authors:** Martin Lindow, Mårten J. Tyrberg, Veikko Pelto-Piri, Anna Björkdahl

**Affiliations:** 1 Department of Medical Sciences, Uppsala University, Uppsala, Sweden; 2 Centre for Clinical Research Västmanland, Uppsala University, Västerås, Sweden; 3 Department of Psychology, Uppsala University, Uppsala, Sweden; 4 University Health Care Research Center, Faculty of Medicine and Health, Örebro University, Örebro, Sweden; 5 Centre for Psychiatry Research, Department of Clinical Neuroscience, Karolinska Institutet, & Stockholm Health Care Services, Region Stockholm, Sweden

**Keywords:** Psychiatrist, Quality improvement, Implementation, Violence prevention, Safewards, Engagement, Coercion.

## Abstract

**Background:**

Restrictive practices, such as seclusion and restraint, in psychiatric inpatient settings carry significant risks of harm and raise critical ethical concerns, which has prompted efforts to minimize their use. Models like Safewards, with its ten interventions, have shown promise in reducing conflict and containment but require active engagement from all healthcare professionals. Despite their leadership roles, psychiatrists’ engagement in implementing Safewards remains underexplored, even though their involvement is likely critical for the model’s success. This study aimed to identify key facilitators and barriers to psychiatrists’ engagement in implementing Safewards and other quality improvement work.

**Methods:**

In this qualitative exploratory study, semi-structured interviews and inductive content analysis were utilized. Ten psychiatrists from nine psychiatric clinics in Sweden, providing both voluntary and involuntary care and implementing Safewards to varying extents, were recruited via convenience sampling. Participants, equally distributed by gender, had an average of 12 years of experience in their roles. Interviews were conducted in person or digitally, lasting 30–90 min, and transcribed verbatim. Data were analyzed using qualitative content analysis, with coding and categorization conducted collaboratively to ensure consistency. Reflexive practice and the COREQ checklist were applied to enhance trustworthiness.

**Results:**

Psychiatrists’ engagement in Safewards and quality improvement efforts was influenced by factors tied to their professional role and the clinical work environment. Positive influences included leadership aspects, professional training, and visible benefits for patient care, such as improved communication with patients and staff. Barriers included a narrow care perspective, feelings of detachment from holistic patient care, and the unpredictable nature of psychiatrists’ work. Time allocation, prioritization, and support from local management also played crucial roles in shaping engagement.

**Conclusions:**

This study identifies key facilitators and barriers to psychiatrists’ engagement in implementing Safewards, offering guidance for enhancing their participation. Strengthening leadership, broadening perspectives, and ensuring protected time for quality improvement initiatives may optimize multidisciplinary collaboration. Future research should examine whether increased psychiatrist participation positively affects Safewards outcomes.

**Trial registration:**

Clinical trial number: not applicable.

**Supplementary Information:**

The online version contains supplementary material available at 10.1186/s12888-025-07058-x.

## Background

In psychiatric inpatient care, patients often experience severe psychological distress, which can lead to behaviors where staff need to support and sometimes restrict the patient. However, restrictive practices, including the use of coercive measures—such as seclusion, restraint, or forced medication—carries significant risks of injury and trauma and raise critical ethical concerns [1–3]. Minimizing violence and the use of restrictive practices in mental health services has therefore been set as a top priority by the World Health Organization [4]. In psychiatric inpatient settings, the commitment of all healthcare professionals to focus on prevention and heightened ethical awareness is required to achieve this goal [5]. It involves a nuanced balancing act between delivering person-centered, trauma-informed care whilst maintaining safety for all during acute and potentially high-risk situations. In recent years, models aiming at reducing restrictive practices, such as Six Core Strategies and Safewards, have been developed and implemented in psychiatric settings with promising results [6–8]. While the psychiatrist is responsible for decisions regarding coercive measures, all healthcare professionals are obliged, through the International Code of Medical Ethics formulated by the World Medical Association [9], to strive to prevent or minimize harm for patients, to not engage in degrading practices and to respect the rights and decisions of patients, including those with intellectual, cognitive, and psychosocial disabilities. Two United Nations declarations, the Convention on the Rights of Persons with Disabilities [10] and the Convention Against Torture and Other Cruel, Inhuman, or Degrading Treatment or Punishment [11], have clarified and strengthened the human rights of patients in psychiatry, and put strong demands on member states. Efforts toward human rights-based mental health policies and services have thus been recognized as a global imperative, as emphasized by a report by the Special Rapporteur transmitted to the UN Human Rights Council in 2017 [12]. Furthermore, exploring and promoting alternatives to coercion in mental health care through active engagement in quality improvement efforts should, according to a Position Statement by the World Psychiatric Association [13], be regarded as a fundamental aspect of psychiatrists’ professional role.

Quality in healthcare has been defined as care that is safe, effective, patient-centered, timely, efficient, and equitable [14]. Similarly, quality improvement refers to the continuous effort by all stakeholders to create changes that enhance patient health, the care provided, and the professional learning environment [15]. According to Silver et al. [16], understanding the contextual factors that enable or hinder quality improvement is fundamental and requires acknowledging aspects such as management support, motivation to change, physician involvement and team diversity. To maximize available resources and unlock the full potential of a healthcare organization, ongoing quality improvement is essential. Obtaining such benefits requires, in addition to time and resource investment, that the quality improvement is sustained over time [16]. According to Batalden et al. [15], it is crucial for all healthcare professionals to recognize that they essentially have two roles: performing their regular duties and continuously striving to improve them. The required level of involvement can be challenging for those in professions that do not typically engage in quality improvement as part of their routine work, such as physicians. To increase the engagement of physicians, Batalden et al. [15] argue that it is critical for healthcare leadership to create connections between quality improvement goals and processes. Similarly, Kaplan et al. suggest that it is also important to foster a culture supportive of quality improvement [17]. Alongside supportive leadership, studies have shown that frontline staff, including physicians, with their firsthand observations play a vital role in identifying areas that require improvement and suggesting necessary changes [18, 19]. Thus, engaging physicians is essential for any healthcare organization aiming to improve quality [20]. Moreover, when physicians are involved in areas such as strategic planning and organizing patient care, positive effects on patient outcomes and improvements in physician performance have been documented [21–23]. Snell et al. [24] have described physician engagement as “the experience that some physicians have in being interested in the quality of their workplace and being motivated to take an active leadership role in helping improve it”. It has been identified as a key factor in ensuring the long-term success of quality improvement within healthcare organizations [19], and a randomized controlled trial by Berner et al. [25] found physician leadership to be highly associated with successful quality improvement initiatives. However, many physicians do not actively participate in these efforts, partly due to the traditional physician role, which is characterized by strong independence and control over clinical decisions. Over the past few decades, this role has been challenged by management and other authorities seeking to exert more control over clinical work, often driven by cost-efficiency concerns. Rundall et al. [26] found in a survey study that this shift has led to skepticism and distrust among physicians toward management’s motives for change, with physicians prioritizing such initiatives that align more with clinical outcomes over management’s financial goals. According to Deilkås et al. [27], a significant factor influencing physician engagement is the time allocated for quality improvement in their schedules. Their research showed that when physicians were given time for such work, they were more likely to engage out of their own interest. Other factors influencing engagement include professional role, workplace hierarchies, training, and managerial support. The idea that time constraints, rather than a lack of motivation, are the main barrier to physician engagement is supported by studies by Allwood et al. [28]. Moreover, a supportive work environment that values professional autonomy has been suggested to boost physician engagement by providing opportunities to develop leadership skills. Conversely, when managers fail to acknowledge or value physician leadership, it can hinder engagement [24, 29, 30]. Other approaches that have been suggested by Spurgeon et al. [31] to increase physician engagement, include employing more physicians and providing appropriate compensation, thereby creating both economic incentives and opportunities for professional development. Similarly, efforts have been made to develop concrete strategies for physician engagement [29, 32], which emphasize the importance of ensuring sufficient staffing so that physicians’ time is not overly occupied by other duties. Studies have shown that when physicians are engaged in quality improvement initiatives, they can help foster a culture of change that impacts many levels of healthcare organizations [17, 33]. At the same time, Davies et al. [34] found that limited knowledge of quality improvement methods, differing perspectives on what constitutes high-quality care, and the belief that high-quality care is already being provided could have a negative impact on physicians’ engagement.

Based on the literature, the involvement of physicians in quality improvement work related to minimizing violence and coercion appears critical. Safewards is a comprehensive model aimed at reducing conflict and containment in psychiatric and mental health inpatient settings. Since its launch in 2014, it has spread rapidly across the globe and has been translated into eleven languages. Grounded in empirical research, the model takes a holistic approach by addressing multiple factors contributing to the escalation of violence in inpatient environments. The model is regarded as highly context-dependent, including ten interrelated interventions that collectively influence the behaviors of both patients and staff [35, 36]. The ten interventions are outlined in Table 1.

**Table 1:** The 10 Safewards interventions as described by fletcher et al. [37]

Intervention	Description
Clear mutual expectations	Patients and staff work together to create mutually agreed aspirations that apply to both groups equally
Soft words	Staff take great care with their tone and use of collaborative language. Staff reduce the limits faced by patients, create flexible options, and use respect if limit setting is unavoidable
Talk down	De-escalation process focuses on clarifying issues and finding solutions together. Staff maintain self-control, respect, and empathy
Positive words	Staff say something positive in handover about each patient. Staff use psychological explanations to describe challenging actions
Bad news mitigation	Staff understand, proactively plan for, and mitigate the effects of bad news received by patients
Know each other	Patients and staff share some personal interests and ideas with each other, displayed in unit common areas
Mutual help meetings	Patients offer and receive mutual help and support through a daily, shared meeting
Calm down methods	Staff support patients to draw on their strengths and use/learn coping skills before the use of PRN medication or containment
Reassurance	Staff touch base with every patient after every conflict on the unit and debrief as required
Discharge messages	Before discharge, patients leave messages of hope for other patients on a display in the unit

The Safewards model emphasizes the importance of understanding not only patient behaviors but also staff practices, the ward’s physical and social environment, and the external factors that may influence patients’ mental well-being. By addressing these key areas, the model aims at fostering a more supportive and therapeutic ward atmosphere, which is crucial in preventing conflict (e.g., aggression, threats) that might otherwise lead to containment measures including coercion (e.g., seclusion, restraint) [3]. Several studies have demonstrated positive outcomes, such as reductions in conflicts and containment measures, as well as improvements in ward culture [3, 7, 38–40]. However, in psychiatric inpatient care, making such changes to a ward culture is rarely a one-person effort. Therefore, a core aspect of the Safewards model is its emphasis on social inclusion, where both staff and patients work together as co-creators of a shared, non-violent environment. According to Safewards, this participatory approach empowers patients, fosters therapeutic relationships, and reduces the power imbalances that can often drive conflict. Another key feature of the Safewards model is its focus on language. It recognizes that how staff communicate with patients and each other can either escalate or de-escalate stress and conflict. By using positive, calming, and non-confrontational language, Safewards stands apart from more traditional, authoritarian approaches to violence prevention, which often emphasize control and physical interventions [2, 3]. Additionally, Safewards has been associated with increased staff job satisfaction, reduced workplace stress, and an enhanced sense of safety due to its interventions. Patients have also reported feeling more respected and heard, as the model emphasizes open communication, trust-building, and conflict de-escalation [41].

However, challenges remain in the implementation of Safewards and maintaining adherence to the model over time can be difficult [39]. Another challenge is gaining full staff buy-in, underscoring the need for ongoing training and support to ensure effective implementation. Without sustained staff commitment, the consistency of Safewards interventions may diminish, potentially reducing their long-term effectiveness [40]. Given the complex nature of Safewards, it is vital to consider different organizational levels when planning and implementing its interventions. In their systematic review, Björkdahl et al. [36] describe the external health system level as comprising societal, external factors, such as laws and policies, that influence the health system. The organizational level is defined as focusing on internal aspects such as priorities, budgets, and other inherent organizational mechanisms. At the local level, various actors influence the implementation of Safewards on a particular ward, with management, local formal and informal leaders, and staff each playing their part in this complex process. In psychiatric inpatient settings, the implementation of Safewards and other violence prevention interventions are therefore closely intertwined, requiring active engagement from all relevant healthcare professionals [5, 42]. Psychiatrists, as specialized physicians with established leadership roles in both clinical care and management, play a critical role not only on the wards but also within the broader healthcare organization, often influencing patient care as well as local policies and priorities. Patients and ward staff rely heavily on psychiatrists’ decisions concerning treatment, leave, discharge, legal matters, and their participation in meetings with patients, families, and the care team. Consequently, it is reasonable to assume that psychiatrists’ engagement, or lack thereof, in supporting and implementing Safewards training and interventions would significantly impact the model’s outcomes. Furthermore, given the communicative nature of psychiatrists’ work, where difficult messages are regularly conveyed to patients, engaging in Safewards interventions such as *Bad news mitigation*, *Soft words* or *Calm down methods* may be seen as a natural extension of their role. However, published literature that evaluates Safewards often overlooks or only briefly mentions the perspective of physicians, including psychiatrists. In the aforementioned systematic review by Björkdahl et al. [36] ten such studies that explored the perspectives of various groups, such as staff members, Safewards leads, champions, managers, and patients were included, but none specifically examined the views of psychiatrists. A notable exception is a study by Baumgardt et al. [43], in which psychiatrists took part in leading the implementation of Safewards on two locked wards. The experience and commitment of one psychiatrist, in particular, were identified as facilitating the implementation process. This gap, however, highlights the need to further investigate psychiatrists’ engagement in Safewards to promote different strategies to enhance their involvement. The aim of this study, therefore, was to identify key facilitators and barriers to psychiatrists’ engagement in implementing Safewards and other quality improvement work in psychiatric inpatient settings.

## Methods

### Design

The study was conducted using a qualitative exploratory research design, with semi-structured individual interviews as the data collection method and qualitative content analysis for data interpretation [44].

### Settings and sample

Ten psychiatrists were recruited using a convenience sampling method from nine different psychiatric clinics in Sweden. These clinics provided both voluntary and involuntary care and had implemented Safewards to varying extents. The selection of clinics was diverse, including five general psychiatric clinics, one forensic psychiatric clinic, one psychiatric addiction center, and one child and adolescent psychiatric clinic. The psychiatrists—five male and five female—were known to have experience in psychiatric settings where Safewards had been implemented. They were contacted via email by AB and VP and invited to participate in the study. After receiving information about the study, all approached psychiatrists agreed to participate and provided written informed consent. The psychiatrists included in the study had an average of 12.4 (SD = 8.7) years of experience in their current roles, ranging from 1 to 30 years.

### Interview guide and procedure

A semi-structured interview guide was used, including questions about experiences with Safewards and quality improvement work in general (Additional file 1). The guide was not pilot-tested prior to the study. Single-session interviews were conducted either in person or digitally via Microsoft Teams. All interviews were conducted by ML, who had no prior relationship with the participants. Interviews were conducted with only ML and the participant present, except for one instance where two participants from the same clinic were interviewed together. The interviews typically lasted around 60 min, ranging from approximately 30 min to 90 min. The digitally recorded audio material was transcribed verbatim, producing an average of about ten pages of text per interview.

### Analysis and interpretation

The transcribed interviews were analyzed using inductive qualitative content analysis, following the recommendations of Elo et al. [44]. Both ML and AB separately read through the interviews multiple times to familiarize themselves with the data. During the organizing phase, recurring content patterns of whole sentences and sometimes smaller sections of text, marked as meaning units, were identified, and labeled with preliminary codes. This was done separately by ML and AB for about half of the interview texts. After each round, ML and AB discussed the coding until they reached consensus regarding the selected units of meaning and the applied codes [45]. Next, ML independently proceeded with coding the entire dataset. Unclear data segments were discussed between ML and AB until consensus on the coding was reached. The coded segments were then organized into categories and sub-categories by ML, and was finalized with the help of AB and MT. The full analysis was conducted in the participants’ and authors’ mother tongue (Swedish) and translated afterwards.

To enhance trustworthiness, the research team applied reflexive practice throughout the study [46]. This included maintaining an ongoing dialogue to critically examine how their own perspectives, including their professional backgrounds and preconceptions, might influence the interpretation of the data, the selection of interview citations and the overall writing of the study. Additionally, the COREQ checklist was applied to ensure thorough reporting of the research [47].

## Results

In this study, it was clear that both knowledge of Safewards and involvement in its implementation varied significantly between the informants, some of whom participated actively in Safewards-related work, while others took a more passive stance. A combination of several factors, collectively described here as *physicians' professional role*, was found to significantly influence their engagement in Safewards and quality improvement work in general. Elements such as a sense of leadership, professional training, and the opportunity to demonstrate competence positively affected their commitment to this work. However, a narrow perspective on care and a sense of alienation from holistic patient care hindered engagement. Additional factors influencing psychiatrists’ participation were related to the *clinical work environment*, a category collectively describing both work environment for healthcare professionals, and care environment for patients. Many participants recognized clear benefits for patient care resulting from Safewards, which motivated them to take part in its implementation. The model also appeared to positively impact the communication between psychiatrists and patients, as well as between psychiatrists and other staff members. Time allocation in psychiatrists’ schedules and their ability to prioritize were also important, along with support from local management. A major barrier to engagement was the unpredictable nature of psychiatrists’ work, which created a sense of detachment from specific wards or clinics. An overview of categories and sub-categories is presented in Table 2.

**Table 2:** Categories and sub-categories of identified factors

Categories	Sub-categories
Physicians’ professional role	Sense of leadership
	Specialized expertise and growth
	Clinical detachment from holistic patient care
Clinical work environment	General positive effects
	Collaborative practice and communication
	Organizational support and resource allocation
	Workplace displacement

### Physicians' professional role

#### Sense of leadership

*“We definitely have a leadership role*,* so somehow we also need to take on that role and develop it”* (Participant 2).

Many participants highlighted a strong sense of leadership as a motivator for engaging in quality improvement work, a notion rooted in their professional role, established through years of medical training and often lengthy careers. There was a perceived responsibility tied to this role, with the belief that psychiatrists, owing to their elevated hierarchical position, should dictate the conditions on the ward, including the direction of quality improvement efforts. Striving to facilitate such initiatives was, according to some participants, viewed as an essential aspect of the work of senior psychiatrists in particular.

*“Work in the ward is led by physicians*,* and especially the senior physician sets the tone*,* so if that person has a way of working that involves quality improvement*,* it needs to diffuse so every professional category understands that’s how we should work”* (Participant 1).

Participants noted that senior psychiatrists may have an educational responsibility, particularly in being clear about their views on quality improvement work, allowing other professional categories to follow their lead. This concept of the psychiatrist as a central figure within the care team was frequently emphasized, with participants arguing that psychiatrists should contribute to the team’s quality improvement efforts, at least in theory. They suggested that this competence could be applied in various contexts, such as workplace meetings, where psychiatrists collaborate with nursing staff to organize patient care.

*“To me*,* the physician is a central figure in the care team*,* so in that sense I think it’s a given that you really have a lot to contribute*,* also around quality”* (Participant 6).

One key aspect of leadership discussed by participants was the need to acknowledge and accept the leadership role, which was seen as crucial for a psychiatrist’s ability to fulfill the associated responsibilities. Most participants described leadership not as an inherent part of the psychiatrist’s role, but as something that one must grow into gradually, often driven by increasing clinical responsibilities. Additionally, some participants expressed a sense of duty to continuously develop their leadership skills, even if they had not actively sought the leadership role. Taking on leadership within the care team was framed as both a personal expectation and one from the broader care environment.

#### Specialized expertise and growth

Some participants noted that a psychiatrist’s specialized expertise, which goes beyond leadership, could significantly impact various aspects of quality improvement work, including enhancing staff well-being. Several participants felt that Safewards had the potential to improve staff well-being, and many participants expressed a sense of responsibility and competence to contribute to this process. Moreover, it was highlighted that psychiatrists, through strong emphasis on teamwork in their training, have a natural inclination toward quality improvement and that early-career doctors or residents often engage in small or larger projects aimed at quality improvement as part of their professional development.

*“I’ve always been that person who encourages colleagues to do such* [quality improvement] *work*,* and I’ve supervised several resident physicians in such work”* (Participant 7).

Several participants described Safewards as a concept that is easily accessible and therefore appealing to less experienced physicians who might feel they have limited medical expertise to offer patients. Safewards could to them provide an opportunity to contribute to patient care without requiring extensive prior experience, thus promoting professional growth.

*“It’s* [Safewards] *been of greater use particularly for younger and less experienced colleagues*,* to feel more useful so to say*, [like] *‘It’s something I can work with’. Otherwise*,* there is a risk of becoming more passive toward the patient and sort of not believing that one can contribute much as being new and inexperienced”* (Participant 1).

Paradoxically, however, almost none of the participants recalled regularly discussing Safewards with colleagues or medical students. Despite this, Safewards was emphasized as aligning with core aspects of psychiatrists’ professional roles, including the drive for quality improvement, communication skills, and the promotion of health for both patients and staff.

#### Clinical detachment from holistic patient care

A significant barrier to engagement in Safewards and quality improvement work in general was the perception, shared by several participants, that tasks primarily focused on direct patient care or nursing interventions were not relevant to psychiatrists. Traditional physician and nurse roles, along with ward hierarchies, were sometimes lifted in support of this view. Some participants described themselves as detached from routine patient care, stating they had “other problems” to address beyond managing ‘difficult patients’. Instead, other staff categories were viewed as more suited to perform the practical, ward-based tasks that Safewards involves.

*“The further you are from the care itself*,* for example we* [psychiatrists] *are the least updated*,* then nurses a little bit better*,* but assistant nurses are even better because those are the ones that do it in practice”* (Participant 2).

Some participants described that both psychiatrists and other staff could perceive that the psychiatrist’s role was not essential for Safewards. This perception sometimes resulted in psychiatrists being excluded from discussions about Safewards or not proactively engaging in them. Although the physician’s leadership role was recognized by both psychiatrists and staff, it still often led to a passive stance toward involvement with Safewards.

*“But I feel that it’s not unnatural for them* [nursing staff] *that I’m not taking part in any concrete way*,* that me being there as moral support is enough. That’s how I’ve perceived it”* (Participant 6).

To some participants, Safewards was viewed as a parallel process occurring behind the scenes, a process that psychiatrists were not naturally invited to join. Consequently, although most participants had a good understanding of the Safewards initiatives on their ward or in the clinic, some had not given it much thought or were even unaware of Safewards at their workplace.

*“Spontaneously right now I feel*,* like I said*,* when I got the e-mail*,* I thought “Oh*,* we have Safewards?“. It’s about on that level”* (Participant 10).

Several participants described Safewards as a more multifaceted approach to patient care, with psychiatrists’ lack of engagement in Safewards highlighting their clinical detachment from this type of mutual staff-patient preventive care. At the same time, many participants said they valued a patient-oriented perspective, which they saw as both a strength and a motivation to engage in Safewards. This contrast was explored by several participants, who argued that emphasizing the diagnosis and treatment of the individual patient carries the risk of losing sight of the broader context. Some participants suggested that psychiatrists may not fully consider the needs of the patient community on the ward as a whole. In doing so, important aspects of both individual and group perspectives could be overlooked, which some participants felt otherwise came naturally in the context of Safewards.

*“I think that the physician’s process is very linearly orientated*,* firstly you think in terms of diagnostics and then when you think of treatment you always think “what is to be done and how can we evaluate the effect of what is being done?“*,* while perhaps not really seeing the patient’s way through all this”* (Participant 5).

Several participants noted that psychiatrists’ focus on the care and treatment of individual patients, rather than the collective patient population, can pose challenges for quality improvement initiatives like Safewards.

### Clinical work environment

#### General positive effects

Participants generally held a positive attitude toward Safewards and quality improvement work overall, describing several key benefits for both the work- and care environment. They observed that implementing Safewards on the ward improved patient well-being and supported recovery. Other noted positive outcomes included a reduction in negative incidents on the ward, an increased likelihood of staff reporting such incidents, and a decrease in staff sickness leave. Participants also highlighted Safewards’ role in enhancing predictability and safety on the ward.

*“I think that* [Safewards] *has promoted closeness between patients and staff*,* and if staff feel more confident with the tools that they’ve got to use*,* then it’s much easier to approach and talk to patients that may be perceived as unpleasant.* […] *So*,* I believe that has especially helped assistant nurses to feel more secure in their role”* (Participant 1).

The implementation of Safewards on the wards was generally seen as fostering a more positive atmosphere among both patients and staff. Key elements contributing to this positive change included a sense of working collaboratively toward shared goals, an interest in Safewards itself, and the addition of something new and engaging to the workplace. Conversely, some participants suggested that Safewards did not add much beyond already established routines. While they acknowledged its benefits to patient care, they felt that Safewards was just a different way of labeling practices that had long been in place.

*“And this is perhaps the most common objection when it comes to both person-centered care and even this project*,* that those physicians that are a bit skeptical in nature may say ‘but isn’t much of this obvious?’”* (Participant 5).

While some participants viewed Safewards as a natural way of working, others saw it as a system and a structured platform for effective patient care. In this way, several participants valued Safewards as a reliable framework, especially helpful when managing negative events on the ward, contrasting with others who saw it as somewhat redundant.

*“Yes*,* it may seem obvious when talking about* [Safewards] *but being able to apply it systematically in a pressured situation isn’t as obvious*,* and you only need to step into any ward to convince yourself of that”* (Participant 5).

Positive effects from the implementation of Safewards and other quality improvement initiatives were consistently highlighted by participants as significantly motivating their engagement in such work. While the tools provided through Safewards were not always viewed as entirely new methods, participants nonetheless reported an increased sense of security and positive cultural shifts on the wards, that promoted well-being for all.

#### Collaborative practice and communication

A major benefit of Safewards, which motivated the participants’ engagement, was its facilitation of communication with both patients and staff. Participants noted this advantage while observing staff interactions with patients and during their own meetings with patients, such as when delivering bad news or other difficult information. Several participants described how they valued reminders from staff regarding Safewards-related communication strategies.

*“Every word counts. Safewards is the tool that we as physicians can use to improve our art of communicating with the patients”* (Participant 4).

One participant described it as ‘gaining a common language’ for interacting with both patients and staff, noting that this also enhanced collaborative efforts. Having committed staff to facilitate quality improvement work was appreciated by participants but was also recognized as something that could not always be taken for granted. Some participants described that the competence of staff, including training and ethical values, influenced and encouraged their own inclination to engage in Safewards and quality improvement initiatives. However, several participants were somewhat pessimistic about consistently keeping and recruiting staff with the qualities needed to drive meaningful care improvements.

*“Of course*,* it would have facilitated the quality improvement work to have staff that really cared and wanted to raise the quality as well”* (Participant 3).

Some participants expressed frustration over feeling excluded from discussions about Safewards, noting that collaborative practice and communication on the ward were not always effective. In those situations, some participants described how psychiatrists might resort to focusing on other, often clinical, aspects of their work, which could risk further miscommunication and deepen the divide between professional groups, as described by a senior participant:

*“At least I haven’t*,* what shall I say*,* taken part in any discussion*,* and I don’t know when those discussions have taken place. Because you’re much closer to the clinical work when you’re on the ward”* (Participant 2).

While almost none of the participants reported actively seeking to investigate the causes of these issues or attempting to solve them, there was a general view that enhanced communication and collaboration between professional groups was desirable.

*“And that’s why I believe that it’s good that we also know what they* [the staff] *are working on*,* because then maybe we too can motivate patients or help with this work”* (Participant 9).

#### Organizational support and resource allocation

Several participants highlighted the importance of support from ward managers for their engagement in quality improvement work, particularly in relation to Safewards. In some instances, ward managers played a significant role as leaders in the implementation process by providing psychiatrists with the necessary resources to participate in Safewards. This could involve organizing regular meetings on the ward to discuss relevant topics or reminding psychiatrists and other staff of the various aspects of Safewards. Additionally, senior psychiatric managers could inspire a culture of quality improvement by their own engagement.

*“My immediate manager is medically responsible for this ward*,* and she is very interested in quality and in having a holistic view of the patient*,* and that things are being done properly”* (Participant 3).

While most participants did not have specific time designated for quality improvement in their ordinary workday, some enjoyed a certain degree of flexibility in their schedules, making it easier for them to set aside time. Additionally, the flexibility of colleagues in covering for each other’s absences in everyday clinical work was another important factor that facilitated these activities. However, most participants described their workloads as cluttered, with numerous tasks and competing interests, leaving little room for commitment to Safewards.

*“I feel that*,* considering our workload*,* we have no time for* [Safewards], *especially during that period”* (Participant 9).

Some participants highlighted their own ability to prioritize as an important factor in ensuring that quality improvement work was not neglected. Several participants viewed quality improvement as an ethical obligation, not something optional or open to question. Instead, they argued that ensuring and enhancing quality must be prioritized, regardless of resource scarcity.

*“And I believe that quality improvement work is such an obvious cornerstone in all that one does that we can’t just deflect and say that “we would love to devote ourselves to quality if only we had more money or more time”*,* because then we’ve started in the wrong end”* (Participant 5).

Overall, the participants emphasized the importance of having a structured organizational framework and support for psychiatrists to engage in Safewards and quality improvement work in general. This included having a manager who understands the nature the psychiatrists’ work and the specific challenges they face. They also noted that when resources, including time, were scarce, the ability to prioritize and be flexible became even more essential.

#### Workplace displacement

Many participants perceived engagement in quality improvement work as a long-term commitment. The unstable and unpredictable work environment faced by psychiatrists was however seen as particularly disruptive to such commitment. Participants noted that the psychiatrist workforce was often understaffed, leading to a small representation within the overall workgroup and difficulties in collaborating with other groups of staff on quality improvement initiatives. Additionally, the frequent reassignment of psychiatrists from one workgroup or ward to another, often with little notice, contributed to an atmosphere of uncertainty, which participants described made them less inclined to engage in quality improvement work.

*“It’s a problem that we as physicians move around so much*,* since healthcare is so divided*,* and you really don’t get stable placement somewhere until you’re a specialist. It took me 18 years from when I started studying until I got a permanent employment*,* which is completely crazy! So*,* you don’t feel at home anywhere*,* and then you don’t feel like doing any quality improvement work either”* (Participant 3).

The lack of a sense of belonging was described as alienating psychiatrists from actively participating in the team-based work of patient care, which could be influenced by Safewards or other quality improvement initiatives. This disconnect was particularly evident when participants discussed the contrast between a traditional psychiatrist focus on diagnostics and treatment and the holistic approach to patient care promoted by Safewards. According to the participants, this tension contributed to their frustration, as they struggled to keep a detailed medical focus on a patient’s illness and recovery while simultaneously feeling obligated to possess knowledge of nearly every aspect of the patient’s well-being.

*“It’s not as clear to everyone*,* I think*,* that you are a motor in a team when there are several others who take part in the patient’s care who have more contact with the patient than you have.* […] *So*,* in that sense you become more of a stand-in character while at the same time*,* as a physician*,* you should have a holistic perspective”* (Participant 5).

Several participants expressed that by the time they became aware of Safewards on the ward, the implementation process was already well underway, making it somewhat difficult or unnatural for them to join in. This was further reinforced when workplace meetings addressing Safewards were sometimes held without inviting psychiatrists to participate. This led to frustration among psychiatrists and added to the uncertainty of their work environment.

*“You don’t even get summoned to workplace meetings. “Yes*,* we’re having a workplace meeting*,* but you don’t belong to this workplace*,* so you don’t need to participate”.* […] *I find that to be extremely frustrating and painful”* (Participant 3).

The frequent workplace reassignments and the resulting sense of not belonging were highlighted as major obstacles for psychiatrists striving to engage in quality improvement work. While some participants appeared indifferent to this reality, many expressed their frustrations and a sense of resignation.

## Discussion

The results suggest that psychiatrists could play an important role in the successful implementation of Safewards. While Safewards could be used as a multidisciplinary model to minimize conflict and containment, our findings indicate that its relevance and uptake among psychiatrists are influenced by a range of aspects including structural, cultural, and individual factors. Notably, the results underscore the well-known importance of leadership in driving physician engagement [24]. Participants expressed that psychiatrists, who most often enjoy leadership roles, could influence their peers through advocacy and example. Whether this could include acting as champions for Safewards, or improving Safewards training, is still unclear, but the importance of opinion leaders in normalizing new practices is well-established [30]. Furthermore, leveraging psychiatrists’ involvement at various organizational levels could potentially facilitate the successful implementation of Safewards [36]. However, according to our findings, integrating this approach into the current clinical work environment of psychiatrists remains a challenge. Therefore, expanding psychiatrists’ leadership roles to include quality improvement, rather than being almost exclusively centered on clinical duties appeared in our study to be an important aspect. Broadening the traditional scope of psychiatrists’ roles and perspectives on patient care to adopt a more holistic approach could also be recommended, as another recurring aspect in our results was the perception that Safewards aligns more closely with traditional nursing roles, which creates a sense of disconnect for psychiatrists. It is possible that this perception stems from the model’s emphasis on practical, relational strategies that are less commonly associated with traditional medical roles. Highlighting the scientific evidence base that Safewards is built upon, which shows the effectiveness of relational strategies to minimize conflicts and enhance safety for both patients and the multidisciplinary team on inpatient wards, could possibly make it more relatable to psychiatrists’ clinical priorities. This would align with prior research showing that physicians engage more readily with initiatives they perceive as directly relevant to their core responsibilities [26]. Moreover, reminding physicians of their ethical obligations in psychiatric care and treatment and presenting Safewards or similar preventive models early in their careers, such as during medical school or residency training, could help foster familiarity with these types of interventions and reduce resistance. Accordingly, early exposure to quality improvement initiatives, before learners become “desensitized to any dysfunctional aspects of the health system”, could be beneficial, as expressed by Brown et al. [48]. Similarly, a particularly novel finding in our study was the potential for Safewards to engage early-career physicians who may feel less confident in their roles. For these individuals, Safewards provides a structured framework to contribute to patient care, ward safety, and interprofessional collaboration, potentially fostering professional growth and confidence. This finding could open new pathways for targeted interventions aimed at this professional group.

The WPA Position Statement [13] on psychiatrists’ responsibility to explore alternatives to coercion served as the ethical foundation for this study. The Safewards model represents an effort to transform psychiatric and mental health inpatient settings into human rights-based services, a view that was shared, albeit implicitly, by participants in this study focusing on psychiatrists’ experiences with Safewards. While the participants generally highlighted the interventions of Safewards as useful and meaningful to patient care and outcomes, interprofessional communication, and safety on the wards, some considered it as merely a new way of labeling such work that was already taking place. This coincides with other research showing that the implementation of Safewards can be considered by staff as common practice [36], which in itself can hinder further engagement [34]. It was in this study, however, evident that these potential benefits of Safewards should be regularly highlighted and discussed to maintain psychiatrists’ engagement. To achieve this, many aspects of psychiatrists’ work could potentially be framed within the various Safewards interventions, as illustrated in this study, where psychiatrists and staff at times collaboratively made effective use of Safewards as a communication tool. Furthermore, for psychiatrists to be motivated to participate, they need to feel like valued members of the team, with consistent support from both management and administration to ensure there is time, flexibility, and authority to engage in such work. This aligns with findings from both Allwood et al. [28] and Deilkås et al. [27], who point at several such influencing factors. Finally, stable working conditions, as highlighted in previous research [29–32], are essential for psychiatrists to sustain long-term commitment to Safewards. The different barriers to psychiatrists’ engagement in Safewards that were identified in this study, e.g., time constraints, competing priorities, and unstable work environments, reflect structural challenges in the implementation of quality improvement efforts. To address these and fully leverage existing psychiatrist engagement, organizations could draw on findings from studies like this for guidance in adopting strategies to promote change.

### Strengths and limitations

The aim of this study was to explore psychiatrists’ engagement in Safewards within psychiatric inpatient settings, examining the factors that influence their engagement and disengagement in implementing Safewards and quality improvement initiatives in general. In this sense, the study was novel, as it delved into largely uncharted territory, addressing an issue that is often overlooked in similar research. Although several influencing factors were identified, we had hoped for more concrete findings regarding actual participation in Safewards or other prominent quality improvement efforts. Nonetheless, we believe the findings are important, as they highlight several obstacles psychiatrists must overcome to engage in quality improvement work. A limitation is the use of convenience sampling that, with a relatively small number of participants, could increase the risk of selection bias and limited applicability of the findings to other settings. Focusing exclusively on psychiatrists’ own perception of their engagement in the implementation of Safewards, while excluding the perspectives of other professional groups, could also be considered a limitation. However, it is a strength that participants were drawn from a diverse range of psychiatric clinics and that all approached psychiatrists agreed to participate in the study. Another strength lies in the comparative coding process between coders, with key decisions made through consensus.

## Conclusions

This study provides insights into the facilitators and barriers to psychiatrists’ engagement in the implementation of Safewards and other quality improvement work, offering guidance for organizations aiming to enhance psychiatrists’ participation in such efforts. Strengthening psychiatrists’ leadership, broadening their perspectives, and providing them with protected time to engage in quality improvement initiatives could more effectively leverage the potential for successful multidisciplinary collaboration through initiatives like Safewards. Future research should investigate whether strong participation by psychiatrists positively impacts Safewards outcomes.

## Electronic supplementary material

Below is the link to the electronic supplementary material.

Supplementary Material 1

## Data Availability

The datasets generated and/or analyzed during the current study are not publicly available due to ethical issues involving participants’ data and privacy.

## Abbreviations

COREQ: Consolidated Criteria for Reporting Qualitative research
UN: United Nations
PRN: Pro re nata. A medication prescribed to treat short term or intermittent medical conditions, not to be taken regularly
WPA: World Psychiatric Association
