# Supplementary material for: Psychiatrists’ experiences with the implementation of safewards and other quality improvement work: an explorative, qualitative interview study
Source: BMC Psychiatry. 2025 Jun 11;25:593. doi: 10.1186/s12888-025-07058-x (PMC12153107; doi:10.1186/s12888-025-07058-x)
Supplement: Supplementary file 1 — Supplementary Material 1 [file 12888_2025_7058_MOESM1_ESM.docx]

# Psychiatrists’ Experiences with the Implementation of Safewards and other Quality Improvement Work: An Explorative, Qualitative Interview Study

# Interview guide

**Introduction**

- The interviewer introduces themselves and any observer/other researcher.
  Briefly explain the purpose of the study:
  - That we are interviewing physicians who, in various ways, are or have been connected to units involved in the Safewards project.
  - That the study focuses on physicians' experiences and thoughts on quality improvement work/projects, both in general and in relation to Safewards.
- Provide assurance of confidentiality and explain how the material will be handled – recording, transcription, reporting, and confidentiality.
- Offer the opportunity to ask questions, then ask participants to sign the consent form.

**Demographics**

- Could you briefly tell us a little about yourself?
  - Are you a specialist physician/psychiatrist or not?
  - What is your professional role (junior doctor, senior consultant, MAL, other)?
  - How many years have you worked as a physician in psychiatry?
  - How long have you been working at your current workplace?

**Quality Improvement Work**

A common definition of healthcare quality is care that is safe, effective, user-inclusive, continuous and coordinated, appropriate, and equitably distributed.

1. What is your perception of physicians' general attitude toward quality improvement work?
2. What is your personal attitude toward such work? **(**For MALs**:** How do you view quality improvement work in your role as MAL?)
3. In what way do you engage in quality improvement work, if at all?
4. Do you have previous experience with quality improvement work in healthcare? Outside of healthcare?
5. Do you feel that you have the practical conditions in your professional role to participate in quality improvement work? Could you elaborate?
6. What specific practical conditions would you wish for in order to (further) engage in such work?
7. Do you feel that your opinions on quality improvement work are valued at your workplace? How?
8. Do you have any other thoughts on the physician’s role in quality improvement work? The relationship with other professional groups? Hierarchical structures? Competence?

**Implementation and Effects of Safewards**

1. Have you been involved in the implementation or any other part of the Safewards project? How? Why not?
2. a. Have you discussed Safewards with other physicians/students? With whom? In what way? What kind of response did you receive?
3. b. Which aspects of Safewards do you, as a physician, find most relevant for psychiatric inpatient wards?
4. How do you perceive the extent to which Safewards is used in your workplace?
5. What does your current engagement with Safewards look like? In general? Any specific interventions?
6. How is Safewards being used by the staff? In general? Any specific interventions?
7. How is Safewards being used by patients? In general? Any specific interventions?
8. Do you see any ways in which the implementation of Safewards could have been made easier? How?
9. Can you identify any factors that have hindered the implementation of Safewards? Examples?
10. Do you see any positive effects of Safewards? In general? For you personally?
11. Do you see any negative effects of Safewards? In general? For you personally?
12. Has Safewards had any impact on relationships, particularly between patients and staff, on the ward? Examples?

**Summary and Conclusion**

- If possible, provide a summary and/or key reflections from the interview and ask the participant whether they agree with the summary and if there is anything they would like to add.
- Thank the participant and ask if they have any further questions about the study.
